# Combination of Preoperative Circulating Tumor Cell Count and Neutrophil-Lymphocyte Ratio for Prognostic Prediction in Hepatocellular Carcinoma Patients after Curative Hepatectomy

**DOI:** 10.1155/2022/7305953

**Published:** 2022-07-16

**Authors:** Hang-Hang Ni, Xi-Hua Yang, Cheng-Lei Yang, Qian Zhang, Jing-Xuan Xu, Lu-Nan Qi, Bang-De Xiang

**Affiliations:** ^1^Department of Hepatobiliary Surgery, Guangxi Medical University Cancer Hospital, Nanning, China; ^2^Key Laboratory of Early Prevention and Treatment for Regional High-Frequency Tumors, Ministry of Education, Nanning, China; ^3^Department of Gastrointestinal Surgical Oncology, Chenzhou No. 1 People's Hospital, Chenzhou, 423000 Hunan, China; ^4^Guangxi Liver Cancer Diagnosis and Treatment Engineering and Technology Research Center, Nanning, China

## Abstract

**Background:**

Both the preoperative neutrophil-lymphocyte ratio (NLR) and circulating tumor cell count (CTC) are associated with poor prognosis in hepatocellular carcinoma (HCC). The purpose of this study was to explore the prognostic value of these two indices (CTC-NLR) in HCC.

**Methods:**

We retrospectively collected demographic and clinical data, including NLR and CTC, from 97 patients with HCC who underwent curative hepatectomy at our institution from March 2014 to May 2017. X-Tile software was used to confirm the optimal cut-off value of NLR and CTC for predicting overall survival (OS) in this study. OS were also analyzed using Kaplan-Meier and Cox regression methods. Based on preoperative CTC and NLR, patients were divided into three groups: CTC-NLR (0), CTC-NLR (1), and CTC-NLR (2). Relationships of CTC-NLR with clinicopathological factors and survival were evaluated.

**Results:**

Preoperatively, CTC positively correlated with NLR. Patients with NLR and CTC higher than the cut-offs had shorter OS than patients with low NLR and CTC. Kaplan-Meier analysis, and log-rank tests revealed significantly lower OS among patients with CTC-NLR scores of 0, 1, and 2. Uni- and multivariate analyses showed that CTC-NLR (hazard ratio 2.050, *P* = 0.005), CTC (hazard ratio 2.285, *P* = 0.032), and NLR (hazard ratio 1.902, *P* = 0.048) were independent predictor of OS. A time-dependent ROC curve indicated that the prognostic efficacy of the CTC-NLR at 1 year (0.714) was better than that of NLR (0.687) and CTC (0.590); the prognostic efficacy of the CTC-NLR at 2 years (0.746) was better than that of NLR (0.711) and CTC (0.601); the prognostic efficacy of the CTC-NLR at 3 years (0.742) was better than that of NLR (0.694) and CTC (0.629).

**Conclusions:**

HCC patients with higher NLR and CTC tend to show shorter OS. Preoperative CTC-NLR may be associated with poor survival and might be a reliable prognostic predictor in HCC after curative hepatectomy.

## 1. Introduction

Hepatocellular carcinoma (HCC) is one of the most prevalent malignancies in China, and it is associated with a high mortality rate [1]. Although HCC diagnosis and treatment have improved substantially, long-term survival remains unfavorable because of high rates of recurrence and mortality. Conventional prognostic markers for HCC include alpha-fetal protein (AFP), tumor staging, and Barcelona Clinic Liver Cancer (BCLC) staging, but their performance is inconsistent [2]. Therefore, a sensitive and easy-to-measure indicator is urgently required to help predict prognosis in HCC, which may improve patient management and therefore survival.

Circulating tumor cells (CTC) that shed from the primary tumor mass circulate through the bloodstream and travel to different organs, in what is considered a precursor step to metastasis [3–5]. Detecting such cells can serve as a “liquid biopsy” that provides insight into metastasis [4]. Indeed, these cells show potential as a biomarker of HCC progression [6–8]. Similarly, an elevated neutrophil-lymphocyte ratio (NLR) can independently predict overall survival (OS) in patients with HCC after curative hepatectomy [9, 10].

In peripheral blood, CTC interact with inflammatory cells to induce systemic inflammation and recruit neutrophils to premetastatic organs [11], in what is considered a precursor step to distant metastasis. For example, circulating tumor cells can interact with non-malignant cells such as white blood cells to promote metastasis [12] and worsen prognosis [13]. This raises the possibility of predicting prognosis based on counts of CTC. In fact, taking NLR into account in addition to CTC can improve the risk stratification and optimal management of high-risk prostate cancer patients resistant to metastatic castration [14]. Combining NLR with CTC can also improve prognostic prediction in patients with gastrointestinal cancer [15].

This led us to ask whether combining NLR and CTC can improve prognostic stratification of HCC patients. To this end, we reviewed prospective data from HCC patients at our institution and analyzed the association of preoperative CTC and NLR with survival.

## 2. Materials and Methods

### 2.1. Study Population

This study retrospectively enrolled 105 HCC patients treated with R0 resection at the Affiliated Cancer Hospital of Guangxi Medical University in Nanning, China between March 2014 and May 2017. Patients were enrolled if they satisfied all the following inclusion criteria: (1) definitive pathological diagnosis of HCC based on World Health Organization criteria; (2) Child-Pugh A stage and Performance Status Test score of 0–1; (3) no prior anticancer treatment, such as transarterial chemoembolization or radiation; and (4) R0 resection, defined as complete macroscopic removal of the tumor, negative resection margins, and no detectable intra- or extrahepatic metastatic lesions. Relevant clinical and demographic data were obtained for each patient from medical records.

The study was conducted in accordance with the Declaration of Helsinki guidelines, and the study protocol was approved by the Ethics Committee of the Affiliated Cancer Hospital of Guangxi Medical University. On admission, all patients provided written consent for their anonymized medical data to be analyzed and published for research purposes.

### 2.2. NLR and Other Markers

Preoperative blood samples were collected and assayed within one week before surgery. Laboratory measurements included serum albumin (ALB), alpha fetoprotein (AFP), hepatitis B virus DNA (HBV-DNA), total peripheral lymphocyte counts, and total peripheral neutrophils count. Cut-off values were 35 g/L for ALB, 400 ng/mL for AFP, and 5.0 × 10^2^ IU/mL for HBV-DNA, respectively, based on the recommendations of the measuring kit our institute adopted. NLR was calculated as the ratio of the total peripheral lymphocyte count to the total peripheral neutrophil count.

### 2.3. Isolation of Circulating Tumor Cells

The CanPatrol™ system was used to isolate CTCs as previously described [16, 17]. Blood samples were collected one or two days before surgery [16, 18, 19]. Peripheral blood samples (5 mL, anticoagulated with ethylenediaminetetraacetic acid) were collected after discarding the first 2 mL to avoid contamination with skin cells. Red blood cell lysis buffer (SurExam, Guangzhou, China) was used to remove erythrocytes, and the cells were resuspended for 5 min in phosphate-buffered saline (PBS) with 4% formaldehyde (Sigma, St. Louis, MO, USA). Next, the blood was filtered using a system including a filtration tube with an 8 *μ*m pore membrane (Sur Exam), a manifold vacuum plate with valve settings (Sur Exam), an E-Z96 vacuum manifold (Omega, Norcross, GA, USA), and a vacuum pump (Auto Science, Tianjin, China). The pumping pressure was 0.08 MPa [20].

### 2.4. Surveillance and Follow-Up

The 97 patients were followed up every 1–2 months for the first year and every three months thereafter, with a final follow-up date of June 30, 2021. Postoperative follow-up consisted of one or more of the following tests: serum AFP measurement, ultrasonography, dynamic computed tomography, and magnetic resonance imaging. Recurrence was classified as intrahepatic recurrence or extrahepatic metastasis and was based on comprehensive evidence from serum AFP assays, imaging (computed tomography, magnetic resonance imaging, digital subtraction angiography, positron emission tomography-computed tomography), and other examinations. OS was measured by comparing the data of surgery to the data of death.

### 2.5. Statistical Analysis

Statistical analyses were performed with SPSS 25.0 (IBM, Chicago, IL, USA), R version 4.1.2 (http://www.r-project.org/) and GraphPad Prism 8.0 (GraphPad, San Diego, CA, USA). OS was defined as the time from the date of surgery to the date of death for any cause or the last recorded follow-up visit. Patient characteristics were analyzed using descriptive statistics. Intergroup differences were assessed for significance using the chi-squared test. Correlation between NLR and CTC was assessed using Spearman's correlation coefficient. Kaplan-Meier survival curves were calculated and compared using the log-rank test. The optimal cut-off points of CTC and NLR for the OS were calculated using the X-Tile statistical package (version 3.6.1, Yale University, New Haven, CT, USA). X-tile software package was used to generate the optimum cutoff point for continuous variables according to the highest x^2^ value defined by the Kaplan-Meier survival analysis and the log-rank test [21]. X-tile plot shows the presence of significant HCC subpopulations, and a two-dimensional projection of each possible subpopulation was used to show the robustness of the relationship between an outcome and a biomarker. Univariate and multivariate Cox proportional hazard analyses were applied to explore associations between patient characteristics and OS. Differences associated with *P* < 0.05 were considered statistically significant.

## 3. Results

### 3.1. Patient Characteristics and Clinical Outcomes

**Figure 1:**
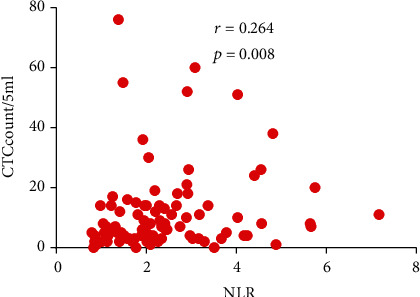
Relationship between preoperative neutrophil-lymphocyte ratio (NLR) and circulating tumor cell count (CTC) in peripheral blood of hepatocellular carcinoma patients.

A total of 97 HCC patients (85 men; mean age, 46.2 years; age range, 20–72 years) who underwent R0 resection were enrolled. During a median follow-up period of 43.0 months (interquartile range (IQR): 11.5–63.0), 46 (47.4%) patients died. Just under half (40, 41.2%) were younger than 45 years, 73 (75.3%) had HBV-DNA levels ≥ 5.0 × 10^2^, 55 (56.7%) had AFP levels ≥ 400 ng/mL, 34 (35.1%) presented multiple tumors, and 42 (43.3%) were in BCLC stages B-C. Patients' demographic and clinical characteristics are summarized in Table 1. Preoperatively, NLR showed a weak positive correlation with CTC (*r* = 0.264, *P* = 0.008; Figure 1).

### 3.2. CTC and NLR

X-Tile was used for traversing expression of the biomarkers and serological indicator value as the cutoff point for dividing the patients and estimating the magnitude CTC and NLR benefits against control in the high- or low-level groups, which were 20 and 2.15, respectively. According to these cut-off values, the high cut-off value was calculated as 1, and the low cut-off value was calculated as 0, CTC-NLR score was calculated as follows: For OS, patients with CTC ≥ 20 and NLR ≥ 2.15 were assigned a score of 2; patients with only CTC ≥ 20 or NLR ≥ 2.15, a score of 1; and patients with CTC < 20 and NLR < 2.15, a score of 0.

### 3.3. Relationship of NLR and CTC with OS

In our cohort, 13 (13.4%) patients had CTC ≥ 20 and 44 (45.3%) had NLR ≥ 2.15. The associations of CTC or NLR with clinicopathological variables are shown in Table 2. CTC was associated with tumor number and BCLC stage. NLR was associated with AFP, tumor number, and BCLC stage.

**Figure 2:**
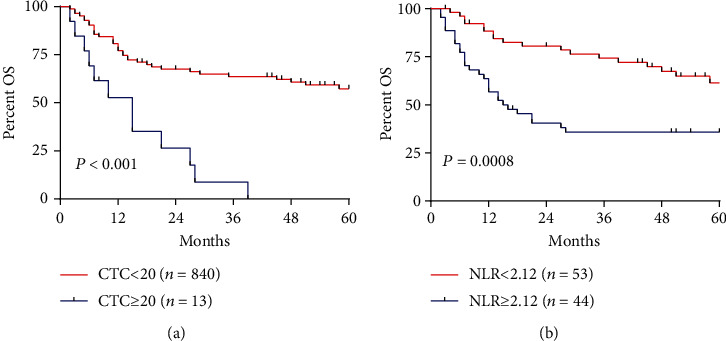
Kaplan-Meier overall survival (OS) curves for patients stratified by preoperative (a) circulating tumor cell count (CTC) or (b) neutrophil-lymphocyte ratio (NLR).

Kaplan-Meier survival curves showed that patients with CTC < 20 had longer OS than patients with higher CTC (Figure 2(a)), similar to those with NLR < 2.15 compared with those with high NLR (Figure 2(b)).

### 3.4. Correlation between Preoperative CTC-NLR Score and Clinicopathological Characteristics

Next, we stratified patients by CTC-NLR score for predicting OS. 49 (50.5%) patients were classified into CTC-NLR (0), 39 (40.2%) into CTC-NLR (1), and 9 (9.3%) into CTC-NLR (2) (Table 3). Our data showed that preoperative CTC-NLR score correlated with AFP, tumor size, tumor number, BCLC stage, and PVTT.

**Figure 3:**
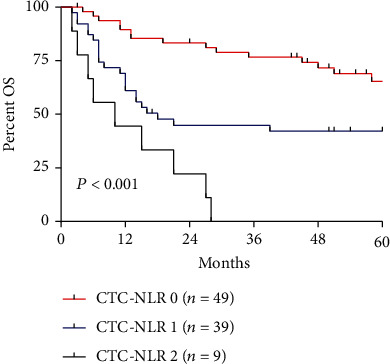
Kaplan-Meier survival curves for HCC patients stratified by preoperative CTC-NLR score for overall survival (OS). Prognostic factors for OS.

Kaplan-Meier curves demonstrated that patients with CTC-NLR (0) were associated with the best OS, whereas patients in CTC-NLR (2) presented the worst OS (Figure 3).

### 3.5. Prognostic Factors for OS

Univariate analysis of OS identified nine pretreatment variables as prognostic factors: CTC-NLR, CTC, NLR, AFP, tumor size, tumor number, BCLC stage, PVTT, and MVI (Table 4).

Factors showing significance by univariate analysis were integrated into multivariate Cox proportional hazard analysis. In this study, NLR, CTC, and CTC-NLR were highly correlated. Therefore, two separate multivariate models were generated to avoid the multicollinearity among the above three variables. In the multivariate analysis, NLR, CTC, and CTC-NLR were independent prognostic factors for shorter OS (Table 5).

### 3.6. Comparison of the Predictive Value of Independent Prognostic Factors

**Figure 4:**
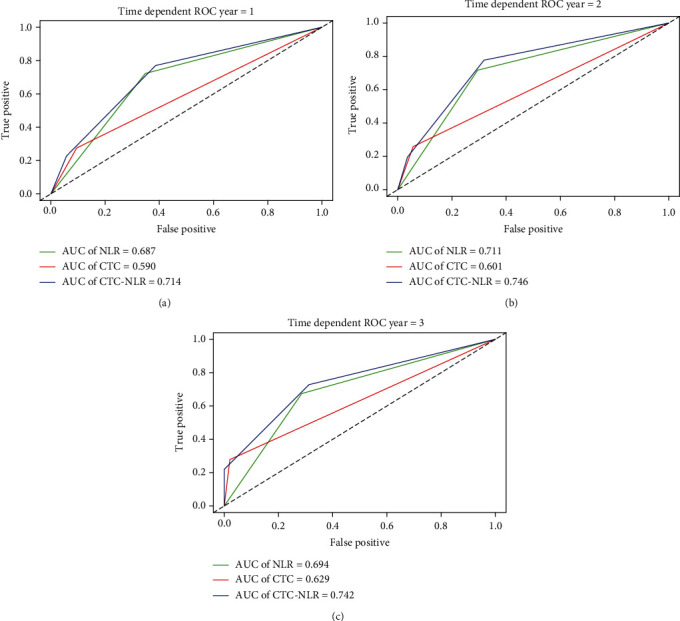
Comparison of the area under the time-dependent receiver operating characteristic curve for overall survival prediction. Comparisons among the inflammation indicators at (a) 1 year, (b) 2 years, and (c) 3 years in patients undergoing partial hepatectomy for hepatocellular carcinoma. ROC: receiver-operating characteristic; NLR: neutrophil-lymphocyte ratio; CTC: circulating tumor cell count.

A time-dependent ROC curve was used to compare the prognostic efficacy of NLR, CTC, and CTC-NLR at 1 year, 2 years, and 3 years. Our results indicated that the prognostic efficacy of the CTC-NLR at 1 year (0.714) was better than that of NLR (0.687) and CTC (0.590) (Figure 4(a)); the prognostic efficacy of the CTC-NLR at 2 years (0.746) was better than that of NLR (0.711) and CTC (0.601) (Figure 4(b)); the prognostic efficacy of the CTC-NLR at 3 years (0.742) was better than that of NLR (0.694) and CTC (0.629) (Figure 4(c)). In addition, the *C*-index of the CTC-NLR was 0.677, which was higher than that of the NLR (0.642) and the CTC (0.591).

## 4. Discussion

Inflammation in the tumor microenvironment, mediated mainly by neutrophils and lymphocytes, can affect tumor development [22, 23]. Neutrophils are the primary source of circulating vascular endothelial growth factor, which is associated with increased risk of recurrence in HCC [24, 25]. Lymphocytes can induce tumor cell death and thereby play an important role in immune surveillance against cancer [26]. In fact, lymphocytopenia may independently predict metastasis in breast cancer [27], and severe lymphopenia may be associated with worse OS in HCC patients receiving radiotherapy [28]. Peritransplant lymphopenia can predict recurrence of HCC after liver transplantation [29]. Therefore, to some extent, the NLR reflects a balance between inflammation and antitumor immunity [30], and alteration of this balance promotes an inflammatory response and facilitates tumor progression. We found that patients with lower preoperative NLR had better OS, consistent with several reports linking NLR to prognosis of HCC patients who underwent hepatectomy [31–35].

CTCs are considered the source of tumor metastasis and recurrence [36, 37]. Several studies have shown that CTCs are independent risk factors for HCC, and patients with higher CTC counts have a poorer prognosis [38, 39]. We also found that patients with lower CTC (<20) had longer OS. CTC may be a useful biomarker of recurrence risk following liver transplantation in HCC patients [40]. These findings likely reflect the role of CTC in metastasis.

We detected a weak positive correlation between preoperative CTC and NLR. This may reflect interactions between neutrophils and circulating tumor cells [41, 42]. As carcinomas develop, patients may be in a systemic inflammatory state in which neutrophils are recruited to organs where circulating tumor cells can invade [11]. We evaluated the combination index CTC-NLR; CTC-NLR score stratified patients into those showing low or high OS. Our results suggest the potential of CTC-NLR score, CTC, and NLR as independent prognostic indicators of OS in HCC. The CTC-NLR had the largest time-dependent AUC in the first to third years. This suggests that the combination CTC and NLR is more effective than CTC or NLR alone in predicting the prognosis of patients with HCC. These results suggest a new avenue for improving risk-stratified management of HCC.

With regard to the mechanisms underlying the relationship between NLR and CTC, they are multifaceted and remain unclear. Noteworthily, recent studies have also shown that the release and survival of CTCs is the result of interactions with the host's immune system, and the number of CTCs in the peripheral blood can also indirectly reflect the immune-nutritional status of patients [13, 43]. Low lymphocyte counts weaken the body's immune defenses, facilitating tumor proliferation, growth, and metastasis [44]. Based on these findings, we deduced that the high NLR reflects the poor immune status of host, which creates favourable conditions for the proliferation, migration, and invasion of tumour cells in tumour lesions, thereby promoting the release of CTCs. Tumor-associated neutrophils (TANs) promote the growth and metastasis of cancer cells through direct effects on cancer cells and indirect effects on tumor cells by changing the tumor microenvironment [45]. In some cases, the fact that CTCs clusters are “the soil with seeds” may help further transfer them to distant organs and continue to grow [13]. This may also be the reason that the preoperative higher CTC-NLR have relationship with multiple tumors and larger tumor size. Meanwhile, the decrease of lymphocyte counts in the peripheral blood makes the CTCs less likely to be attacked by immune cells after entering the bloodstream, so that more CTCs survive and are detected. This also provided a reasonable explanation for the findings of our study on the relationship between high preoperative CTC-NLR and unfavourable clinicopathological features, including BCLC stage and PVTT.

Our study presents several limitations. First, it was a retrospective analysis of patients at a single center. Second, the sample was relatively small, which meant we could not divide patients into separate training and validation cohorts. Third, follow-up was relatively short, and not all patients underwent standardized follow-up procedures according to National Comprehensive Cancer Network guidelines. Therefore, our results should be verified and extended in a future work.

## 5. Conclusions

The present study provides the first evidence that a novel index based on the combination of preoperative CTC and NLR is closely related to postoperative long-term outcomes in surgically treated HCC patients. Preoperative evaluation of the CTC-NLR score may be useful for risk classification and clinical decision-making for HCC patients.

## Figures and Tables

**Table 1:** Demographic and clinical characteristics of the hepatocellular carcinoma patients in the study.

Variable	*n*	%
Sex		
Male	85	87.6
Female	12	12.4
Age (years)		
<45	40	41.2
≥45	57	58.8
HBsAg		
Negative	10	10.3
Positive	87	89.7
Liver cirrhosis		
Negative	4	4.1
Positive	93	95.9
HBV-DNA (IU/mL)		
<5 × 10^2^	24	24.7
≥5 × 10^2^	73	75.3
AFP (ng/mL)		
<400	42	43.3
≥400	55	56.7
ALB (g/L)		
<35	12	12.4
≥35	85	87.6
Tumor size (cm)		
<5	22	22.7
≥5	75	77.3
Tumor number		
1	63	64.9
>1	34	35.1
BCLC stage		
0-A	55	56.7
B-C	42	43.3
PVTT		
Negative	75	77.3
Positive	22	22.7
MVI		
Negative	31	32.0
Positive	66	68.0

AFP: alpha-fetoprotein; ALB: albumin; BCLC stage: Barcelona Clinic Liver Cancer stage; HBsAg: hepatitis B surface antigen; HBV-DNA: hepatitis B virus DNA; HCC: hepatocellular carcinoma; MVI: microvascular invasion; PVTT: portal vein tumor thrombosis.

**Table 2:** Association of preoperative CTC or NLR with clinicopathological variables of HCC patients, based on cut-off values for predicting OS.

Variable	CTC < 20	CTC ≥ 20	*P*	NLR < 2.15	NLR ≥ 2.15	*P*
	*N* = 84	*N* = 13		*N* = 53	*N* = 44	
Sex						
Male	77	12	0.922	45	40	0.559
Female	11	1		8	4	
Age (years)						
<45	35	5	0.827	20	20	0.442
≥45	49	8		33	24	
HBsAg						
Negative	10	0	0.410	4	6	0.518
Positive	74	13		49	38	
Liver cirrhosis						
Negative	4	0	0.957	4	0	0.178
Positive	80	13		49	44	
HBV-DNA (IU/mL)						
<5 × 10^2^	23	1	0.236	15	9	0.373
≥5 × 10^2^	61	12		38	35	
AFP (ng/mL)						
<400	38	4	0.497	32	10	<0.001
≥400	46	9		21	34	
ALB (g/L)						
<35	8	4	0.087	6	6	0.730
≥35	76	9		47	38	
Tumor size (cm)						
<5	20	2	0.750	16	6	0.053
≥5	64	11		37	38	
Tumor number						
1	59	4	0.014	40	23	0.017
>1	25	9		13	21	
BCLC stage						
0-A	52	3	0.020	38	17	0.001
B-C	32	10		15	27	
PVTT						
Negative	67	8	0.144	45	30	0.050
Positive	17	5		8	14	
MVI						
Negative	29	2	0.290	19	12	0.367
Positive	55	11		34	32	

AFP: alpha-fetoprotein; ALB: albumin; BCLC stage: Barcelona Clinic Liver Cancer stage; CTC: circulating tumor cell count; HBsAg: hepatitis B surface antigen; HBV-DNA: hepatitis B virus DNA; HCC: hepatocellular carcinoma; MVI: microvascular invasion; NLR: neutrophil-lymphocyte ratio; OS: overall survival; PVTT: portal vein tumor thrombosis.

**Table 3:** Correlation between CTC-NLR score and clinicopathological characteristics of HCC patients.

Variable	CTC-NLR			
	Score 0 (*N* = 49)	Score 1 (*N* = 39)	Score 2 (*N* = 9)	*P*
Sex				
Male	41	36	8	0.459
Female	8	3	1	
Age (years)				
<45	19	17	4	0.883
≥45	30	22	5	
HBsAg				
Negative	4	6	0	0.204
Positive	45	33	9	
Liver cirrhosis				
Negative	4	0	0	0.060
Positive	45	39	9	
HBV-DNA (IU/mL)				
<5 × 10^2^	14	10	0	0.064
≥5 × 10^2^	35	29	9	
AFP (ng/mL)				
<400	31	8	3	<0.001
≥400	18	31	6	
ALB (g/L)				
<35	4	6	2	0.392
≥35	45	33	7	
Tumor size (cm)				
<5	16	4	2	0.036
≥5	33	35	7	
Tumor number				
1	38	23	2	0.004
>1	11	16	7	
BCLC stage				
0-A	37	16	2	<0.001
B-C	12	23	7	
PVTT				
Negative	43	26	6	0.042
Positive	6	13	3	
MVI				
Negative	19	10	2	0.337
Positive	30	29	7	

AFP: alpha-fetoprotein; ALB: albumin; CTC: circulating tumor cell count; NLR: neutrophil-lymphocyte ratio; BCLC stage: Barcelona Clinic Liver Cancer stage; HBsAg: hepatitis B surface antigen; HBV-DNA: hepatitis B virus DNA; HCC: hepatocellular carcinoma; MVI: microvascular invasion; PVTT: portal vein tumor thrombosis.

**Table 4:** Univariate analysis of clinicopathological characteristics associated with survival.

Variable	Overall survival		
	HR	95% CI	*P*
Sex (M/F)	0.250	0.061-1.034	0.056
Age (≥45)	1.114	0.612-2.027	0.724
HBsAg (positive)	1.226	0.439-3.422	0.697
Liver cirrhosis (positive)	2.570	0.354-18.660	0.351
HBV-DNA (≥5 × 10^2^ IU/mL)	0.850	0.486-1.813	0.850
AFP (≥400 ng/mL)	1.952	1.052-3.624	0.034
CTC-NLR	2.696	1.765-4.119	<0.001
CTC ≥ 20	4.211	2.131-8.321	<0.001
NLR ≥ 2.15	2.632	1.451-4.773	0.001
ALB (≥35 g/L)	0.680	0.304-1.522	0.348
Tumor size (≥5 cm)	4.457	1.595-12.452	0.004
Tumor number (>1)	2.828	1.575-5.075	<0.001
MVI (positive)	8.362	2.984-23.427	<0.001
BCLC stage (B-C)	7.912	4.015-15.592	<0.001
PVTT (positive)	9.005	4.456-18.195	<0.001

AFP: alpha-fetoprotein; ALB: albumin; CTC: circulating tumor cell count; NLR: neutrophil-lymphocyte ratio; BCLC stage: Barcelona Clinic Liver Cancer stage; HBsAg: hepatitis B surface antigen; HBV-DNA: hepatitis B virus DNA; HCC: hepatocellular carcinoma; HR: hazard ratio; MVI: microvascular invasion; PVTT: portal vein tumor thrombosis.

**Table 5:** Multivariate analysis of clinicopathological characteristics associated with survival.

Variable	Overall survival		
	HR	95% CI	*P*
*Model 1*			
CTC ≥ 20	2.285	1.073-4.869	0.032
NLR ≥ 2.15	1.902	1.005-3.600	0.048
AFP (≥400 ng/mL)	0.910	0.466-1.777	0.783
Tumor size (≥5 cm)	1.820	0.612-5.416	0.282
Tumor number (>1)	0.510	0.192-1.351	0.175
MVI (positive)	4.179	1.369-12.756	0.012
BCLC stage (B-C)	4.988	1.430-17.401	0.012
PVTT (positive)	1.831	0.721-4.645	0.203
*Model 2*			
CTC-NLR	2.050	1.235-3.402	0.005
AFP (≥400 ng/mL)	0.884	0.461-1.695	0.710
Tumor size (≥5 cm)	1.831	0.617-5.433	0.276
Tumor number (>1)	0.530	0.205-1.374	0.192
MVI (positive)	4.237	1.393-12.887	0.011
BCLC stage (B-C)	4.865	1.402-16.877	0.013
PVTT (positive)	1.857	0.733-4.700	0.192

AFP: alpha-fetoprotein; ALB: albumin; CTC: circulating tumor cell count; NLR: neutrophil-lymphocyte ratio; BCLC stage: Barcelona Clinic Liver Cancer stage; MVI: microvascular invasion; PVTT: portal vein tumor thrombosis.

## Data Availability

Data is available upon reasonable request from the authors.
